# Synergistic effects of AWD irrigation and potassium application on rice yield and paddy field greenhouse gas emissions

**DOI:** 10.3389/fpls.2026.1840297

**Published:** 2026-06-09

**Authors:** Dandan Wu, Yinghao Li, Zhengyuqi Ma, Shujun Wang, Taotao Chen, Hongtao Zou, Jingjun Li, Yang Gao

**Affiliations:** 1 College of Water Conservancy, Shenyang Agricultural University, Shenyang, China; 2 Postdoctoral Station of Agricultural Resources and Environment, Land and Environment College, Shenyang Agricultural University, Shenyang, China; 3 College of Land and Environment, Shenyang Agricultural University, Shenyang, China; 4 State Key Laboratory of Water Cycle and Water Security, China Institute of Water Resources and Hydropower Research, Beijing, China; 5 Heishan County Key Irrigation Experimental Station, Heishan County Water Conservancy Bureau, Jinzhou, Liaoning, China

**Keywords:** alternate wetting-drying irrigation, global warming potential, greenhouse gas emissions, potassium application, rice yield

## Abstract

**Introduction:**

Irrigation and fertilization are significant for rice productivity and environmental benefits. Evidences on how alternate wetting-drying irrigation (I_AWD_) and potassium (K) together influence greenhouse gas emission (GHG) and rice yield still remain largely unknown.

**Methods:**

A field trial was used to evaluate how K application affect GHG and related microbial functional genes abundance, yield, water use efficiency (WUE), and global warming potential (GWP) under I_AWD_ in 2023 and 2024 in northeast China.

**Results and discussion:**

The results indicated that I_AWD_ significantly enhanced functional genes (*amoA*-AOA, *amoA*-AOB, *nirK* and *nosZ*) abundances, improved *mcrA* gene abundance while reducing that of *pmoA*, and thereby significantly led to rises of 58% (tillering period), 54% (panicle period), and 38% (entire period) in nitrous oxide (N_2_O) emissions and declines of 87% (tillering period), 60% (panicle period), and 76% (entire period) in methane (CH_4_) emissions, averaged in both years. K application significantly reduced the *amoA*-AOA, *amoA*-AOB, *nirK*, and *nosZ* genes abundances, enhanced *mcrA* gene abundance. Compared with K_0_, K_75_ and K_150_ resulted in 14% and 16% reductions in N_2_O emissions, while K_150_ increased CH_4_ emissions by 29%. Relative to I_CF_, I_AWD_ decreased soil NH_4_^+^ concentration and increased soil NO_3_^-^ concentration. Relative to K_0_, K_75_ and K_150_ increased soil NH_4_^+^ concentration and K application did not significantly alter soil NO_3_^-^ concentration. Under continuous flooding irrigation (I_CF_), compared with K_0_, K_150_ significantly increased GWP and greenhouse gas intensity (GHGI) by 28% and 15%, while K_0_ and K_150_ had similar GWP and GHGI under I_AWD_. Compared with K_0_, K_75_ and K_150_ significantly boosted rice yield (8% and 11%) and WUE (15% and 18%), respectively. Principal component analysis indicated that I_AWD_K_75_ was recommended considering environmental sustainability and economic benefit in the paddy ecosystem.

## 1 Introduction

The global average temperature is anticipated to increase by more than 1.5°C in the upcoming 20 years. Rising atmospheric concentrations of greenhouse gases (GHG) methane (CH_4_) and nitrous oxide (N_2_O) is one important cause for global warming (Lou et al., 2024). Rice (*Oryza sativa* L.) is a pillar for global food security and a staple food for billions of people, yet its cultivation imposes a significant environmental challenge (Liu et al., 2026b). Paddy soils contribute to about 33% and 69% of agricultural N_2_O and CH_4_ emissions, respectively (Peng et al., 2025). Therefore, ongoing research focused on identifying management strategies to reduce GHG from paddies while increasing rice yield.

Rice consumes 34%~43% of global agricultural irrigation water worldwide, with continuous flooding irrigation (I_CF_) being a traditional form of water management in rice cultivation system (Poddar et al., 2022). However, I_CF_ has many problems in terms of low water use efficiency, low N utilization efficiency, and high CH_4_ emissions (Zhang et al., 2026). With the increasingly severe water shortage and the rising requirements for environmental sustainability, alternate wetting-drying irrigation (I_AWD_), a mainstream water-conserving irrigation management worldwide has attracted much attention (Xiao et al., 2025; Evangelista et al., 2026). The potential of I_AWD_ significantly reducing irrigation water consumption has been widely demonstrated, and approximately 41% of paddy fields in China are currently irrigated using I_AWD_ (Cheng et al., 2022).

The effects of I_AWD_ on global warming potential (GWP) in paddy fields are reported by many previous researches. CH_4_ emissions typically contribute to a much larger portion of the GWP relative to N_2_O, and this trade-off eliminates the overall GWP reduction effect of I_AWD_ in most cases (Hou et al., 2023; Islam et al., 2022; Li et al., 2024a). Previous research also indicated that the CH_4_ emission decrease was compensated by a N_2_O emission increase, resulting in no significant difference in GWP between I_CF_ and I_AWD_ (Ariani et al., 2022; Echeverría-Progulakis et al., 2025). CH_4_ production requires an anaerobic environment. I_AWD_ significantly reduces CH_4_ emissions by increasing soil oxygen content, which inhibits CH_4_ production and promotes CH_4_ oxidation. However, I_AWD_ cycles promote the soil N metabolic cycle. Since N_2_O is formed during both aerobic ammonia oxidation and anaerobic nitrate reduction processes, I_AWD_ can promote the production and emission of N_2_O. A recent meta-analysis by Zhao et al. (2024), which integrated data from 41 field studies across various climate regions and soil types, found that I_AWD_ significantly reduced CH_4_ emissions by an average of 52% while simultaneously increasing N_2_O emissions by an average of 44% compared to I_CF_, demonstrating a clear trade−off between these two GHG under I_AWD_. N_2_O emission pulses frequently occur during the drying phase of I_AWD_. Lagomarsino et al. (2016) reported that in a Mediterranean rice system, the transition from anaerobic to aerobic soil conditions resulted in the highest N_2_O fluxes under I_AWD_, with fluxes more than doubling compared to I_CF_. If the water layer depth is too thick, N_2_O cannot escape, but the concentration of N_2_O in the soil layer remains relatively high. When the water layer disappears during the drying process, N_2_O is released into the atmosphere, which explains why the N_2_O emission pulse phenomenon occurred. This accumulation release pattern has been further confirmed by a recent work showing that N_2_O predominantly accumulates in the 0~20 cm soil profile under I_AWD_, and that the N_2_O accumulated in the 10~20 cm layer exerts the strongest direct control on surface N_2_O emissions (Liu et al., 2026a). These studies underscore the complex interplay between water regime, soil N transformation processes, and N_2_O emission dynamics under I_AWD_. Another recent global meta-analysis reported that I_AWD_ led to a 43% increase in N_2_O emissions, and reductions in CH_4_ (43%), GWP (37%), and GHGI (39%) (Li et al., 2024a) https://www.sciencedirect.com/science/article/abs/pii/S0378429024003563. The net GWP effect under I_AWD_ is highly site-specific, modulated by climatic conditions and soil properties https://www.sciencedirect.com/science/article/abs/pii/S0378429024003563. Thus, the specific impacts of I_AWD_ on N_2_O and CH_4_ emissions, GWP, and also the underlying mechanisms, are not yet fully understood.

As an essential macronutrient together with N and P for optimal plant growth, K application has long time been overlooked in research (Xiong et al., 2025). The uptake of K by field crops is often consistent with that of N and significantly higher than that of P, and its crucial role in sustaining high yields and improving stress resistance is frequently underscored (Carciochi et al., 2025). Additionally, K exerts a vital influence in regulating N_2_O and CH_4_ emissions via stoichiometric relations with carbon and N (Feng et al., 2021). Under I_CF_, soil anaerobiosis enhances K fixation into clay interlayers and restricts mineral weathering, generally reducing K availability https://ouci.dntb.gov.ua/en/works/lDRXr8g7/. However, I_AWD_ fundamentally alters K dynamics through repeated wetting-drying cycles. In cold region paddy soils of Northeast China, I_AWD_ promotes the accumulation of applied K in the topsoil and facilitates the release of slow release K from soils, whereas I_CF_ exacerbates K leaching (Li et al., 2022b)https://caod.oriprobe.com//articles/44218873/Effect_of_water_management_on_potassium_in_paddy_soil_in_cold_region.htm. The impact of K application on GHG had gradually received attention recently. K application increased N_2_O and CH_4_ emissions, and the results in laboratory experiments were inconsistent with that in field conditions (Li et al., 2024b). Some studies suggested that K fertilization reduced methanogenic bacteria and *nirK* and *norb* denitrifiers abundances, thereby alleviating CH_4_ and N_2_O emissions (Babu et al., 2006; Li et al., 2021). As mentioned above, the impact of K application on GHG emissions and microbial mechanisms governing these processes in paddy field remain unclear. Consequently, in temperate rice growing region, elucidating the mechanisms of K application on GHG and GWP under AWD needs to be done urgently, and a solid foundation for irrigation management and K application in paddy field also needs to be provided.

Previous I_AWD_ interact with nutrient studies have predominantly focused on N fertilizer in combination with I_AWD_. By contrast, the novelty of our study lies in the synergistic effects of K fertilization with I_AWD_, which remains critically unexplored in the existing literature. In this study, we applied a field trial to examine the impacts of K application on N_2_O and CH_4_ emissions and their related functional genes abundance, soil N concentrations, rice yield, GWP, GHG intensity (GHGI), irrigation water, and water use efficiency (WUE) under I_CF_ and I_AWD_. The specific aims of this research: 1) to elucidate the impact of K application on both N_2_O and CH_4_ emissions, and correlate them to changes in the key functional genes abundance (e.g. *amoA*-AOA, *amoA*-AOB, *nirK*, *nirS*, *nosZ*, *mcrA*, and *pmoA*); 2) to identify the impacts of K application on GWP, yield, GHGI and WUE. These results will lay a foundation for stabilizing rice yield and alleviating the environmental burden associated with paddy field production.

## 2 Materials and methods

### 2.1 Field site

A 2-year trial was carried out during 2023 and 2024 at Comprehensive Experimental Base of Water Conservancy, Shenyang Agricultural University (located at 41°49’N, 123°33’E). The experiment station locates in a temperate continental monsoon climate zone. Daily precipitation and mean temperature were represented in **Supplementary Figure 1**. The topsoil (0~20 cm) was silty clay loam and its properties were represented in **Supplementary Table 1**.

### 2.2 Trial design and management

Field trial layout used a split-plot design with 3 replicates. Two irrigation managements (I_CF_, continuously flooding; I_AWD_, alternate wetting-drying) were assigned to the main plot. Three sub-plots of K applications (K_0_, 0 kg ha^–1^; K_75_, 75 kg ha^–1^; K_150_, 150 kg ha^–1^) were set within each main plot. The allocation of the six treatments to the plots within each block was determined using a random number generator (Excel RAND function) prior to the start of the experiment. A water level gauge, a separate water pipeline and a water amount meter were installed in each plot. Soil water potential at 0~15 cm depth was recorded twice a day in each plot using a tensiometer, provided by Institute of Soil Science, Chinese Academy of Sciences. Detailed information on both irrigation managements regarding the water depth and soil water potential thresholds were provided in **Supplementary Table 2**. I_AWD_ had 4 times, once, 4 times, 4 times, and 4 times wetting-drying cycles in the middle tillering stage, late tillering stage, jointing-booting stage, heading-flowering stage, and milky ripening stage, respectively, in 2023. while in 2024, I_AWD_ had 4 times, twice, twice, 3 times, and 3 times wetting-drying cycles, respectively (**Supplementary Figure 2**). Each plot (4 m×3 m) was surrounded using a 40 cm depth PVC border before the trial initiation to inhibit lateral flow of water and nutrients.

Fertilization was split into three times in total, including basal fertilization (1 Jun. 2023 and 30 May 2024), tillering fertilization (15 Jun. 2023 and 20 Jun. 2024) and panicle fertilization (30 Jul. 2023 and 4 Aug. 2024). Urea (46% N, 180 kg ha^–1^), calcium superphosphate (12% P_2_O_5_, 52 kg ha^–1^), and potassium sulfate (50% K_2_O, 150 kg ha^–1^) served as the respective sources of N/P/K. The details of fertilization were as follows: 60% N (108 kg N ha^–1^), 50% K (37.5 and 75 kg K_2_O ha^–1^), and 100% P were used as basal fertilizer; 30% N (54 kg N ha^–1^) was used as tillering fertilizer; and 10% N (18 kg N ha^–1^) and 50% K (37.5 and 75 kg K_2_O ha^–1^) were used as panicle fertilizer. A japonica rice cultivar Liaoxing (*Oryza sativa* L.) was cultivated in both years. Transplanting took place at 1 Jun. 2023 and 30 May 2024, with corresponding harvests at 12 Oct. 2023 and 11 Oct. 2024, respectively. Weeds, insects, and diseases were controlled following conventional agronomic protocols.

### 2.3 Measurements and calculations

#### 2.3.1 CH_4_ and N_2_O monitoring and calculating

Fluxes of CH_4_ and N_2_O were determined by the static closed chamber technique. Each chamber was made up of a transparent acrylic standard chamber, a transparent acrylic extension chamber, and a black PVC based frame. Details regarding the size, installation, and inner components refer to our previous research (Chen et al., 2025b). The based frame positions were alternated between sampling events to avoid repeated disturbance. Samples of gas were taken at 9:00~11:00 am on each sampling day, using a 100-mL syringe at 0, 15, and 30 min following chambers closure. Because most gas emission occurred in the first week after fertilization, gas sampling frequency was every 2 d in the first week post-fertilization, and 7~10 d intervals for the subsequent rice growing periods. Measurements of CH_4_ and N_2_O referred Liu et al. (2024a), and CH_4_ and N_2_O fluxes were computed using Equation (1):

(1) $$F=\rho \times h\times (dC/dt)\times [273/(273+t)]\times p/p_{0}$$

Where *F* is the daily CH_4_ and N_2_O flux (μg m^–2^ h^–1^), *ρ* is CH_4_ and N_2_O density (0.741 and 1.964 kg m^–3^), *h* is the height from ground to the chamber upper edge (m), *dC/dt* is the CH_4_ and N_2_O concentration change rate (μL dm^–3^ h^–1^), *t* is the air temperature in the chamber (°C), *p* and *p*_0_ are chamber air pressure and standard atmosphere pressure (hPa), respectively.

Cumulative CH_4_ and N_2_O emissions were computed by summing all determined fluxes. Missing data between sampling dates were filled by linear interpolation. Cumulative CH_4_ and N_2_O emissions were determined using Equation (2):

(2) $$\text{Cumulative CH}4{\text{ and N}}_{2}\text{O emissions}=\sum_{i=1}^{n}(F_{i}+F_{i-1})/2\times d\times 24\times 10^{-5}$$

Where *F_i_* and *F_i+_*_1_ are adjacent CH_4_ and N_2_O fluxes (μg m^–2^ h^–1^), d is the time interval in two successive measurements.

GWP and GHGI were determined by Equations (3) and (4) (Zhang et al., 2025):

(3) $$\text{GWP}={\text{CH}}_{4}\times 27.9+{\text{N}}_{2}\text{O}\times 273$$

(4) GHGI=GWP/Y

Where GWP and GHGI refer to global warming potential (kg CO_2_ eq ha^−1^) and greenhouse gas intensity (kg CO_2_ eq kg^−1^), respectively. On the 100-year scale, CH_4_ and N_2_O have GWP of 27.9 and 273 relative to CO_2_, respectively. Y (kg ha^−1^) is rice yield.

#### 2.3.2 Soil nitrogen determination and qPCR analysis

Along with each gas sampling, the topsoil (0~20 cm) samples were collected from each plot at three randomly selected points and through mixed before determination. The soil samples were sieved by 2 mm mesh and extracted with 2 mol L^-1^ potassium chloride. The AA3 analyzer (BRAN+LUEBBE, Germany) was used for determining soil NH_4_^+^ and NO_3_^-^ concentrations. Soil samples were combined to form a composite sample during period of high CH_4_ and N_2_O fluxes and from each plot, respectively. Microbial DNA was purified from 0.5 g fresh soil sample with an OMEGA Mag-Bind Soil DNA Kit (M5635-02, USA), and its purity and concentration were determined using Nanodrop spectrophotometer (ND-2000, USA) and 1% agarose gel electrophoresis. The 7 genes abundances (*amoA*-AOA, *amoA*-AOB, *nirK*, *nirS*, *nosZ*, *mcrA* and *pmoA*) associated with CH_4_ and N_2_O emissions were quantified via a qPCR with 16S rRNA gene primers (338F-ACTCCTACGGGAGGCAGCA; 806R-GGACTACHVGGGTWTCTAAT). The AceQ reagent kit qPCR SYBR Green Master (Vazyme, China) was used to conduct the corresponding fluorescence qPCR experiments. **Supplementary Table 3** summarizes the details of gene-specific primers and thermal parameters employed for qPCR. The reaction mixture contained 8 μL 2 × SYBR premixture, along with 0.4 µL each primer (10 μM), 8 µL diluted DNA template. The amplification protocol included 95 °C for 5 min, then 45 cycles of 95 °C for 30 s and 60 °C for 30 s. After the reaction completed, the LightCycler 480 II (Roche, Switzerland) was used for the following analyses. PCR reaction fluorescence was plotted against the cycle number. The cycle where signal intensity crosses the threshold baseline is called the threshold cycle (Ct). The Ct is used for estimating the initial DNA copy number. The copies number (X_0_)was calculated by Equation (5):

(5) $$\text{Ct}=-\text{K}\log{\text{X}}_{0}+b$$

Where *K* and *b* is the slope and intercept of the standard curve, respectively.

The standard curve was constructed on the basis of a 10-fold serial dilution of plasmids that contain the target gene. The amplification efficiency was 85~93%, with R^2^ values of 0.990~0.997.

#### 2.3.3 Rice yield, water use efficiency and economic benefit

At maturity stage, rice plants in three 1 m^2^ area in each plot was selected for manually harvest and allowed to dry naturally at 14% moisture content for yield calculation. Water use efficiency (WUE) was obtained from Equation (6):

(6) WUE=Y/I

Where *Y* is rice yield (kg ha^–1^), and *I* is irrigation volume (m^3^ ha^–1^).

The net ecosystem economic benefit (NEEB) was calculated according to cost of resources input including fertilizer and irrigation, cost of global warming potential and value of rice grain (Li et al., 2022b; Jin et al., 2025), using Equation (7):

(7) $$NEEB=V_{rice}-C_{input}-C_{GWP}$$

Where *V_rice_* is the rice value (CNY2.8 kg^–1^ rice), *C_input_* is the fertilizer and water input cost (CNY1.8 kg^–1^ urea, CNY2.1 kg^–1^ superphosphate, CNY6.5 kg^–1^ K_2_SO_4_, CNY0.16 m^–3^ water), *C_GWP_* is the GWP cost, only considering direct GHG from paddy fields (CNY280 t CO_2_ eq).

### 2.4 Statistical analysis

Analysis of variance (ANOVA) for a split-plot design was done using the R *agricolae* package (version 4.2.0). Non-normally distributed data were log-transformed before ANOVA. Irrigation managements and K applications were designated as fixed factors, with replication considered as the random factor. The whole plot error was used for the main effect (irrigation management), and the subplot error was used for the main effect (K application) and the interaction effect. Tukey’s HSD test was employed for *post hoc* analysis, setting the significance level at *p* < 0.05. The correlations among the functional genes abundance and CH_4_ and N_2_O fluxes peak were analyzed using regression analysis in Origin 2024. Principal component analysis (PCA) was implemented via the *Factoextra* package. Structural equation modeling (SEM) was carried with the *piecewiseSEM* package (Shipley and Douma, 2021). Pathway analysis by SEM was applied to discern the direct and indirect effects of the irrigation management and K application on yield, cumulative N_2_O and CH_4_ emission, and GWP. The adequacy of the piecewise SEMs was evaluated using Fisher’s C test, with a *p*-value exceeding 0.05 denoting acceptable fit (Lefcheck, 2016).

## 3 Results

### 3.1 N_2_O emissions

**Figure 1:**
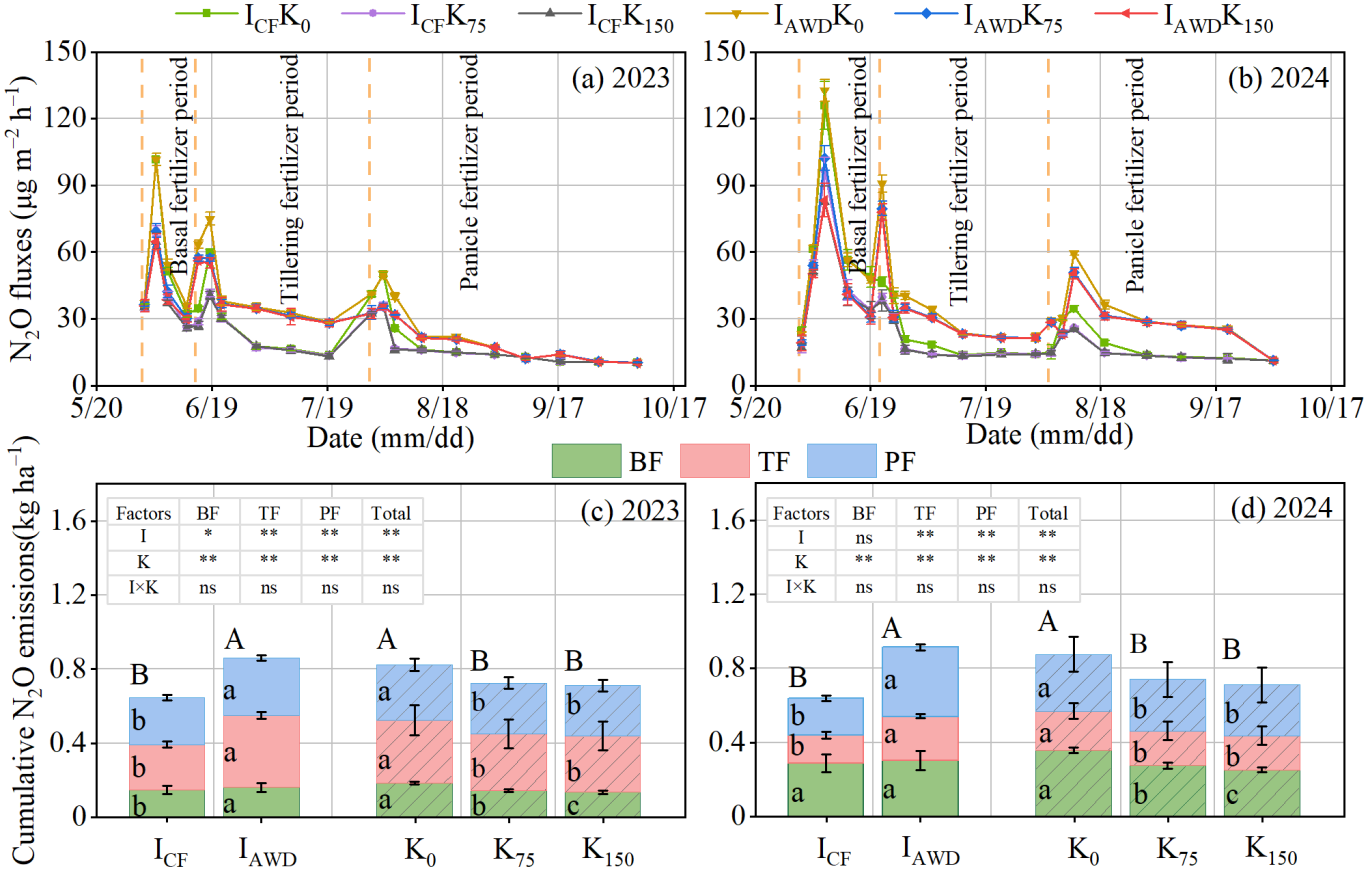
Impacts of irrigation management (I) and potassium application (K) on N_2_O fluxes and cumulative emissions in BP (basal period), TP (tillering period), PP (panicle period), and Total (entire period) in **(A, C)** 2023 and **(B, D)** 2024. Different lowercase letters in column segments indicate significant differences (*p* < 0.05) among treatments at each period, while different uppercase letters above the whole columns indicate significant differences (*p* < 0.05) among treatments at the Total. * and ** indicate significant differences at *p* < 0.05 and *p* < 0.01, respectively. ns indicates non-significant difference. Vertical bars are mean ± standard deviations (*n* = 3).

N_2_O flux presented peak within 7 days and then declined after every fertilization in both years (**Figures 1A, B**). The highest N_2_O fluxes appeared in BP (basal period) within all treatments in both years. I_AWD_ had higher N_2_O flux than I_CF_. The peak of N_2_O flux significantly decreased with the increasing K fertilization during BP. As the growth season progressed, the N_2_O flux peak at K_150_ did not significantly differ with K_75_ at TP (tillering period) and PP (panicle period), but both are significantly lower than K_0_ (**Figures 1A, B**).

Irrigation management significantly influenced cumulative N_2_O emissions at BP, TP, PP, and Total (entire period) in 2023, and TP, PP, and Total in 2024 (**Figures 1C, D**). In comparison to I_CF_, I_AWD_ elevated cumulative N_2_O emissions at BP, TP, PP, and Total by 9%, 59%, 23%, 33% in 2023, and TP, PP, Total by 57%, 85%, 43% in 2024, respectively. Potassium fertilizer significantly influenced cumulative N_2_O emissions at BP, TP, PP, and Total in both 2023 and 2024. Specifically, compared with K_0_, K_75_ and K_150_ caused a reduction in cumulative N_2_O emissions at BP by 22%~26% and 23%~30% in 2023 and 2024, respectively. Relative to K_0_, K_75_ and K_150_ declined cumulative N_2_O emissions at TP (9%~10%), PP (9%~10%), and Total (12%~14%) in 2023, and TP (12%~13%), PP (9%~10%), and Total (15%~19%) in 2024, respectively (**Figures 1C, D**). Moreover, cumulative N_2_O emissions of K_75_ and K_150_ did not differ significantly at TP, PP, and Total over two years.

### 3.2 CH_4_ emissions

**Figure 2:**
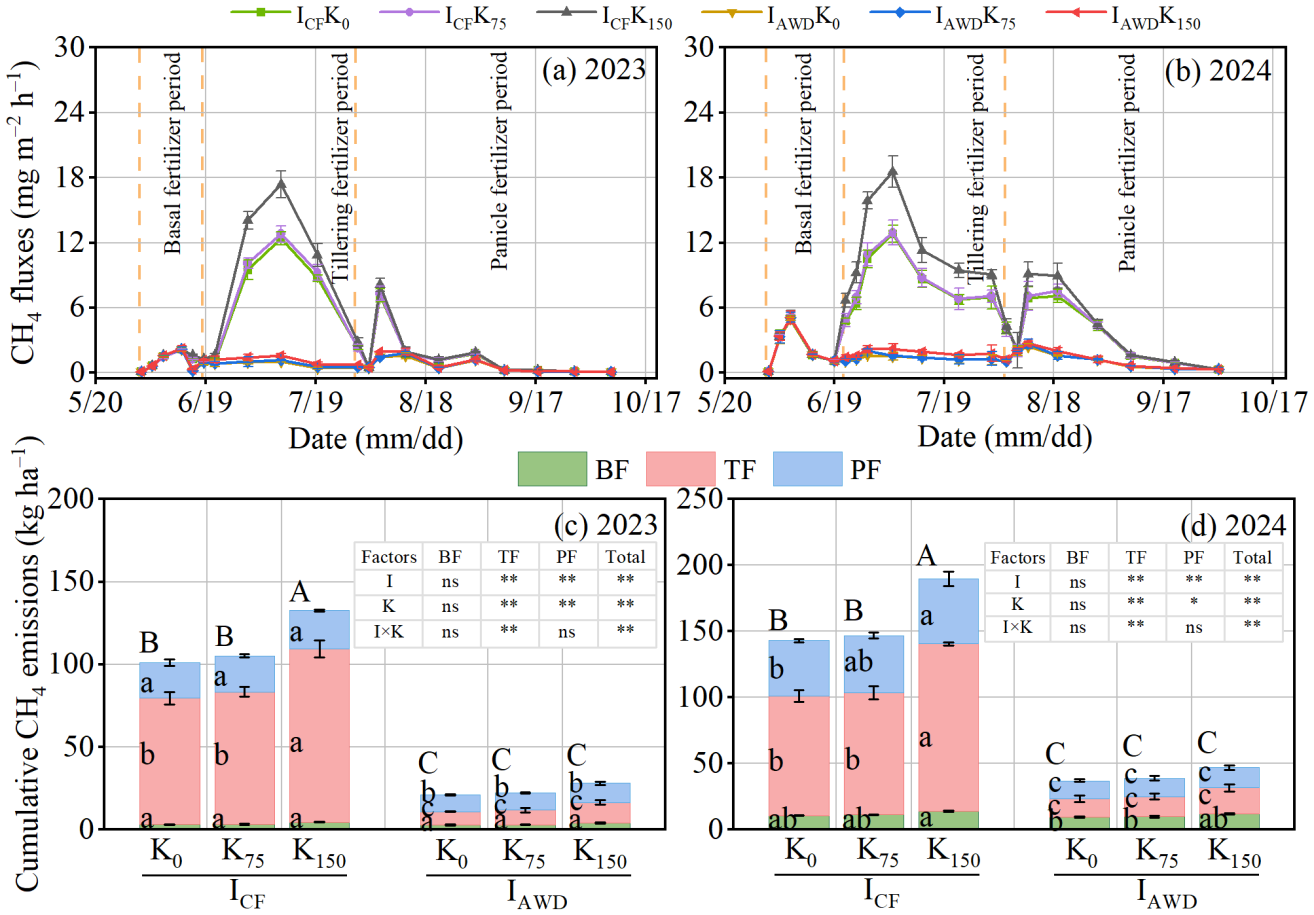
Impacts of irrigation management (I) and potassium application (K) on CH_4_ flux and cumulative emissions in BP (basal period), TP (tillering period), PP (panicle period), and Total (entire period) in **(A, C)** 2023 and **(B, D)** 2024. Different lowercase letters in column segments indicate significant differences (*p* < 0.05) among treatments at each period, while different uppercase letters above the whole columns indicate significant differences (*p* < 0.05) among treatments at the Total. * and ** indicate significant differences at *p* < 0.05 and *p* < 0.01, respectively. ns indicates non-significant difference. Vertical bars are mean ± standard deviations (*n* = 3).

I_AWD_ significantly decreased CH_4_ fluxes peaks compared with I_CF_ at TP and PP in two years (**Figures 2A. B**). As compared to I_CF_, I_AWD_ showed smaller fluctuations of CH_4_ fluxes. The CH_4_ fluxes of I_CF_ had 2~3 peaks at Total in both years. Under I_CF_, K_150_ significantly increased CH_4_ fluxes peak at TP by 40% (10 Jul. 2023) and 45% (5 Jul. 2024), respectively, relative to K_0_. Under I_AWD_, the CH_4_ fluxes were comparable among the three K applications (**Figures 2A, B**).

Irrigation management exerted a significant impact on cumulative CH_4_ emissions at TP, PP, and Total across both experimental years (**Figures 2C, D**). Specifically, compared with I_CF_, I_AWD_ reduced cumulative CH_4_ emissions during TP, PP, Total by 89%, 51%, 78% in 2023, and by 84%, 68%, 74% in 2024, respectively. K application significantly influenced the cumulative CH_4_ emissions at TP, PP, and Total in two years. Relative to K_0_, K_150_ increased cumulative CH_4_ emissions at TP, PP, Total by 39%, 11%, 29% in 2023, and by 40%, 16%, 28% in 2024, respectively. The irrigation and K application interactions were significant at TP and Total both in 2023 and 2024. Under I_CF_, K_150_ increased the cumulative CH_4_ emissions at TP, Total by 37%, 30% (2023) and 40%, 30% (2024), compared to K_0_, respectively. In contrast, under I_AWD_, cumulative CH_4_ emissions did not differ significantly in at TP and Total among the three K applications (**Figures 2C, D**).

### 3.3 Genes abundances related to N_2_O emissions

**Figure 3:**
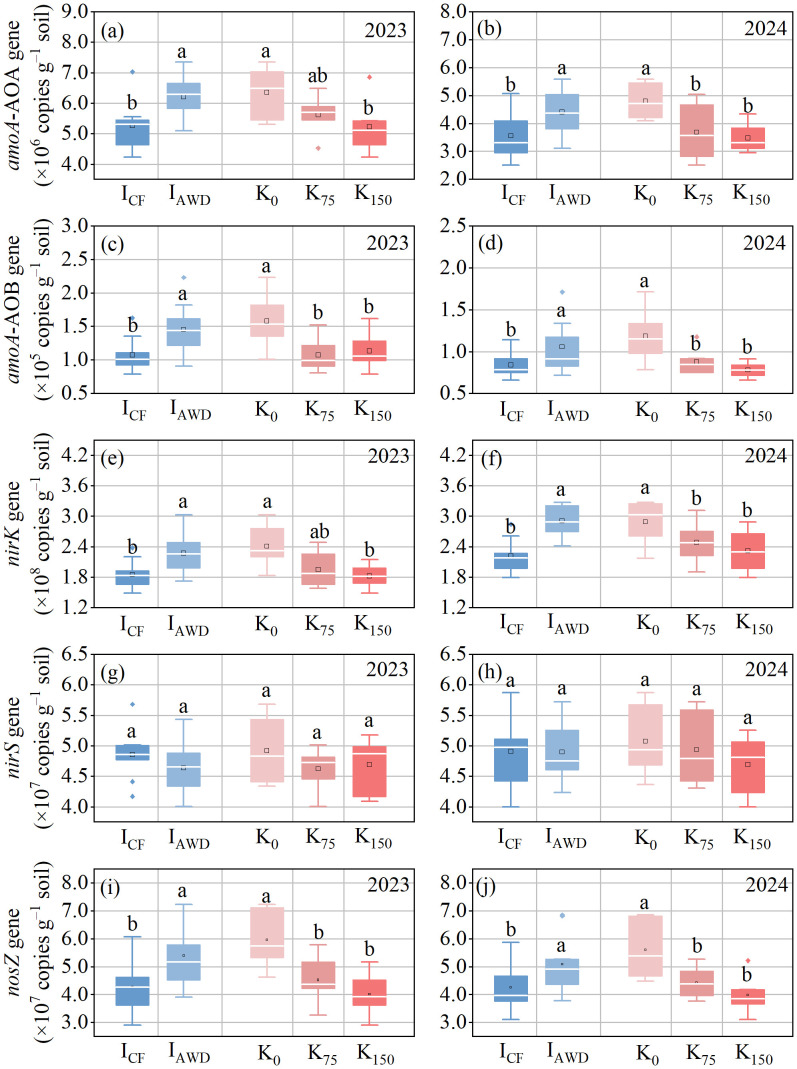
Impacts of irrigation management (I) and potassium application (K) on *amoA*-AOA **(A, B)**, *amoA*-AOB **(C, D)**, *nirK* (e and f), *nirS* **(G, H)**, and *nosZ* **(I, J)** abundances at N_2_O fluxes peak in 2023 and 2024. Different letters above bars indicate significant differences (*p* < 0.05) among treatments.

The abundances of N_2_O related genes were significantly altered by irrigation management in 2023 and 2024 (**Supplementary Table 4**). Compared to I_CF_, I_AWD_ increased *amoA*-AOA, *amoA*-AOB, *nirK*, and *nosZ* abundances by 18%, 35%, 23%, 26% (2023), and 24%, 26%, 31%, 20% (2024), respectively (**Figures 3A–F, I, J**). Irrigation management did not significantly influence *nirS* abundance in either year (**Supplementary Table 4**; **Figures 3G, H**). K application significantly affected *amoA*-AOA, *amoA*-AOB, *nirK*, and *nosZ* abundances in both years (**Supplementary Table 4**). K_150_ decreased *amoA*-AOA, *amoA*-AOB, *nirK*, and *nosZ* abundances by 18%, 28%, 33%, and 24% in 2023, respectively, compared with K_0_ (**Figures 3A, C, E, I**). In 2024, K_75_ and K_150_ decreased gene abundances by 23%~28% (*amoA*-AOA), 26%~34% (*amoA*-AOB), 14%~20% (*nirK*), 21%~29% (*nosZ*), respectively, relative to K_0_ (**Figures 3B, D, F, J**). K application did not significantly influence *nirS* abundance either in 2023 or 2024 (**Supplementary Table 4**; **Figures 3G, H**). The irrigation management and K application interactions on the *amoA*-AOA, *amoA*-AOB, *nirK*, *nirS*, and *nosZ* abundances were not significant over both years (**Supplementary Table 4**).

### 3.4 Genes abundance related to CH_4_ emissions

**Figure 4:**
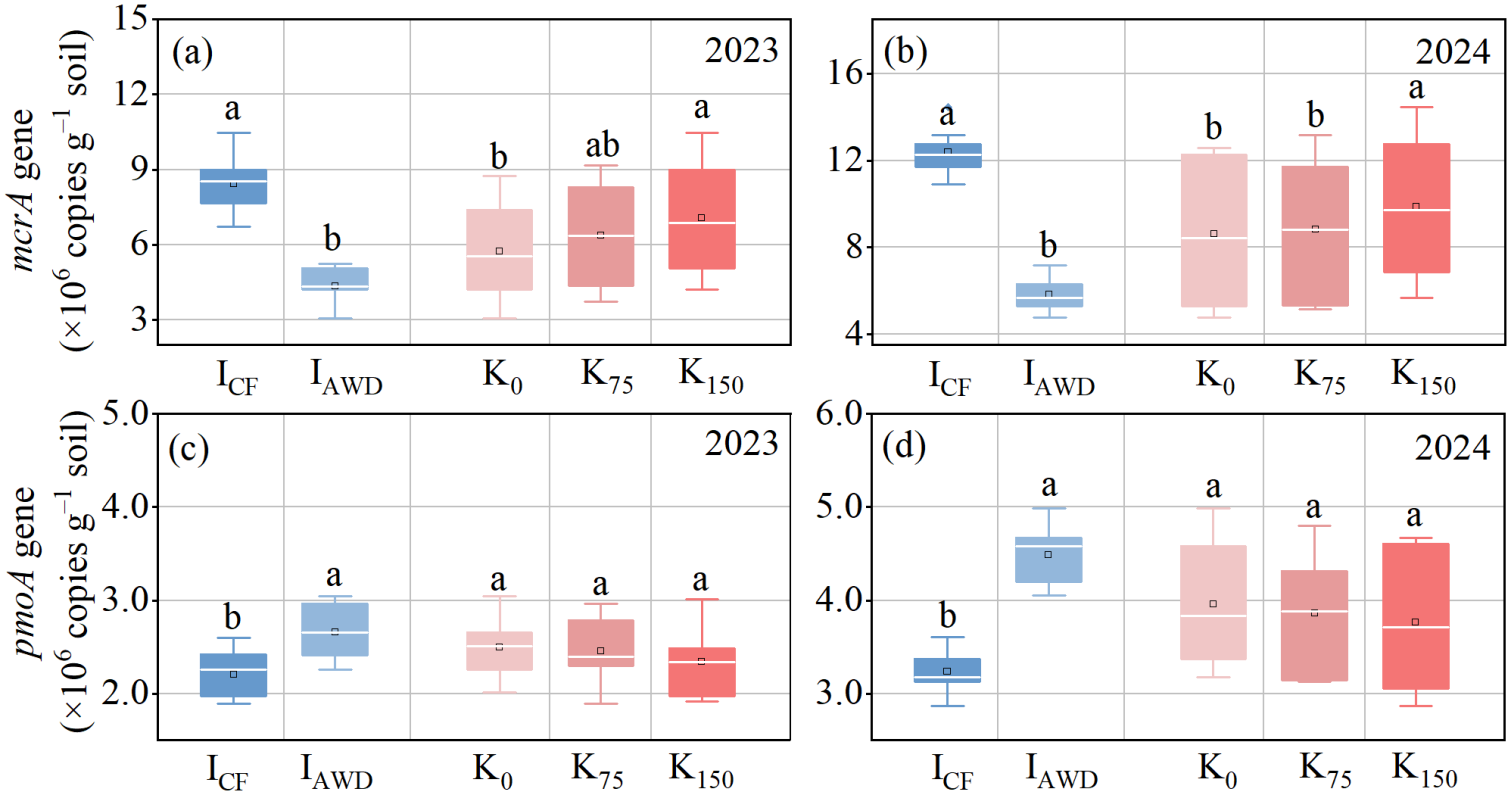
Impacts of irrigation management (I) and potassium application (K) on *mcrA* **(A, B)** and *pmoA* **(C, D)** abundances at CH_4_ fluxes peak in 2023 and 2024. Different letters above bars indicate significant differences (*p* < 0.05) among treatments.

Irrigation management and K application alone significantly affected *mcrA* and *pmoA* abundances, but exerted no significant interactive influence on *mcrA* and *pmoA* abundances in 2023 and 2024 (**Supplementary Table 4**). Relative to I_CF_, I_AWD_ decreased *mcrA* abundance by 48% and 53% in 2023 and 2024, respectively (**Figures 4A, B**). I_AWD_ promoted *pmoA* abundance by 20% and 39% in 2023 and 2024, respectively (**Figures 4C, D**). Relative to K_0_, K_150_ promoted *mcrA* abundance by 23% and 14% in 2023 and 2024, respectively (**Figures 4A, B**). The *mcrA* abundance was comparable between K_75_ and K_150_ in 2023. However, in 2024, K_150_ increased *mcrA* abundance by 12%, relative to K_75_ (**Figures 4A, B**). K application did not significantly affect *pmoA* abundance in 2023 and 2024 (**Supplementary Table 4****;** **Figures 4C, D**).

### 3.5 Soil NH_4_^+^ and NO_3_^-^ concentrations

Irrigation management and K application alone significantly affected soil NH_4_^+^ concentration in TP and PP, but exerted no significant interactive influence on soil NH_4_^+^ concentration in BP, TP and PP in both years. K application significantly affected soil NH_4_^+^ concentration in BP in both years (**Table 1**). Relative to I_CF_, I_AWD_ decreased soil NH_4_^+^ concentration in TP by 27% and 20% and in PP by 17% and 22% in 2023 and 2024, respectively. In BP, relative to K_0_, K_75_ and K_150_ promoted soil NH_4_^+^ concentration by 13% and 24% in 2023, and 12% and 26% in 2024, respectively. In TP, relative to K_0_, K_75_ and K_150_ promoted soil NH_4_^+^ concentration by 14% and 25%, respectively in 2023, and K_150_ promoted soil NH_4_^+^ concentration by 24% in 2024. In PP, relative to K_0_, K_75_ and K_150_ promoted soil NH_4_^+^ concentration by 12% and 17%, respectively in 2023, and K_150_ promoted soil NH_4_^+^ concentration by 15% in 2024.

**Table 1:** Impacts of irrigation management and K application on soil NH_4_^+^ and NO_3_^-^ concentrations in different growth stages in 2023 and 2024.

Main effects	Soil NH_4_^+^ in BP (mg kg^–1^)		Soil NH_4_^+^ in TP (mg kg^–1^)		Soil NH_4_^+^ in PP (mg kg^–1^)		Soil NO_3_^-^ in BP (mg kg^–1^)		Soil NO_3_^-^ in TP (mg kg^–1^)		Soil NO_3_^-^ in PP (mg kg^–1^)	
	2023	2024	2023	2024	2023	2024	2023	2024	2023	2024	2023	2024
I_CF_	35.4a	33.0a	30.3a	27.6a	11.6a	10.4a	2.00a	1.72a	0.992b	0.860b	0.514b	0.405b
I_AWD_	34.0a	31.8a	22.1b	21.9b	9.64b	8.10b	2.09a	1.77a	1.21a	1.08a	0.673a	0.492a
K_0_	30.9c	28.8c	23.2b	22.3b	9.65b	8.57b	2.12a	1.80a	1.15a	1.00a	0.623a	0.472a
K_75_	34.9b	32.1b	26.5a	24.4ab	10.8a	9.39ab	2.05a	1.75a	1.09a	0.964a	0.592a	0.451a
K_150_	38.4a	36.4a	28.9a	27.5a	11.3a	9.82a	1.96a	1.69a	1.07a	0.933a	0.566a	0.422a
ANOVA												
I	ns	ns	*	*	*	*	ns	ns	*	*	*	*
K	**	**	**	**	**	*	ns	ns	ns	ns	ns	ns
I×K	ns	ns	ns	ns	ns	ns	ns	ns	ns	ns	ns	ns

I represents irrigation management, and K represents potassium applications. BP (basal period), TP (tillering period), PP (panicle period). * and ** indicate signiﬁcance level at *p* ≤ 0.05 and *p* ≤ 0.01, respectively. ns indicates non-signiﬁcant.

Irrigation management significantly affected soil NO_3_^-^ concentration in TP and PP in both years. K application and its interactive influence with irrigation management did not significantly affect soil NO_3_^-^ concentration in BP, TP and PP in both years (**Table 1**). Relative to I_CF_, I_AWD_ increased soil NO_3_^-^ concentration in TP by 22% and 25% and in PP by 31% and 22% in 2023 and 2024, respectively.

### 3.6 Regression analysis of CH_4_ and N_2_O emission with microorganism gene abundances

**Figure 5:**
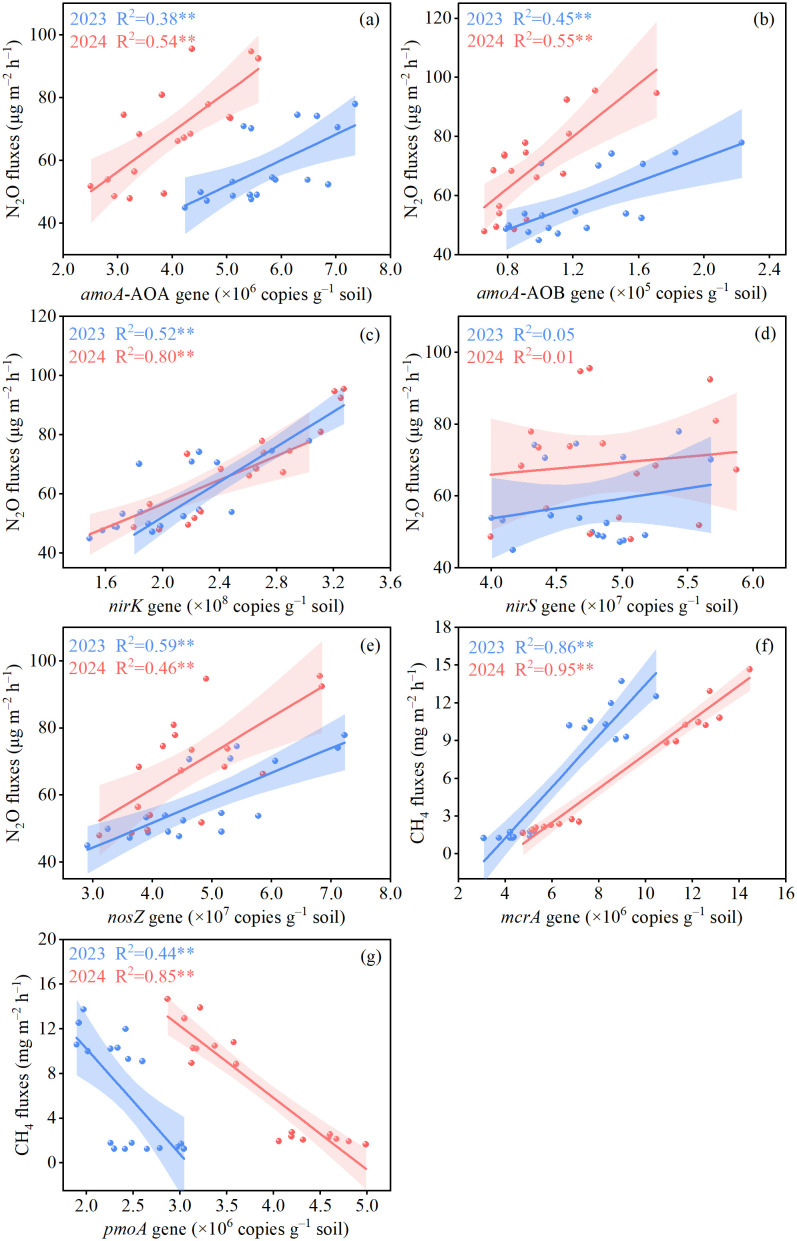
Relationships between CH_4_ and N_2_O fluxes peaks and *amoA*-AOA **(A)**, *amoA*-AOB **(B)**, *nirK* **(C)**, *nirS* **(D)**, *nosZ* **(E)**, *mcrA* **(F)**, and *pmoA* **(G)** abundances in 2023 and 2024. The dashed lines with shades represent the regression lines with 95% confidence intervals at *p* < 0.05.

The linear regressions between the genes abundance and CH_4_ and N_2_O fluxes peak were presented in **Figure 5**. N_2_O flux was significantly positively correlated with *amoA*-AOA (R^2^ = 0.38 and 0.54, *p* < 0.01), *amoA*-AOB (R^2^ = 0.45 and 0.55, *p* < 0.01), *nirK* (R^2^ = 0.52 and 0.80, *p* < 0.01), and *nosZ* (R^2^ = 0.59 and 0.46, *p* < 0.01), in 2023 and 2024, respectively (**Figures 5A–E**). CH_4_ flux was significantly positively correlated with *mcrA* (R^2^ = 0.86 and 0.95, *p* < 0.01), and was significantly negatively correlated with *pmoA* (R^2^ = 0.44 and 0.85, *p* < 0.01) in 2023 and 2024, respectively (**Figures 5F, G**). However, none of the N_2_O fluxes was significantly correlated with *nirS* abundance over the two years.

### 3.7 Yield, NEEB, WUE, GWP, and GHGI

Irrigation management did not significantly alter rice yield, whereas K application significantly affected rice yield in two years (**Table 2**). Compared with K_0_, K_75_ and K_150_ increased yield by 8% and 11% in 2023, respectively, with the same increment observed in 2024. The interaction of irrigation management and K application had no significant effect on rice yield in either year (**Table 2**).

**Table 2:** Impacts of irrigation management and K application on rice yield, irrigation input, net ecosystem economic benefit and WUE in 2023 and 2024.

Main effects	2023				2024			
	Yield (t ha^–1^)	Irrigation (10^3^ m^3^ ha^–1^)	NEEB (CNY ha^–1^)	WUE (kg m^–3^)	Yield (t ha^–1^)	Irrigation (10^3^ m^3^ ha^–1^)	NEEB (CNY ha^–1^)	WUE (kg m^–3^)
I_CF_	7.64a	7.39a	16459a	1.03b	7.56a	6.68a	14805a	1.06b
I_AWD_	7.48a	6.18b	16768a	1.19a	7.36a	5.65b	15315a	1.22a
K_0_	7.12b	7.08a	16374a	1.00b	7.02b	6.37a	14856a	1.03b
K_75_	7.66a	6.69a	17019a	1.14a	7.58a	6.05a	15493a	1.18a
K_150_	7.89a	6.58a	16447a	1.19a	7.77a	6.08a	14831a	1.20a
ANOVA								
I	ns	*	ns	*	ns	*	ns	*
K	**	ns	ns	**	**	ns	ns	**
I×K	ns	ns	ns	ns	ns	ns	ns	ns

I represents irrigation management, and K represents potassium applications. * and ** indicate signiﬁcance level at *p* ≤ 0.05 and *p* ≤ 0.01, respectively. ns indicates non-signiﬁcant. NEEB and WUE represent net ecosystem economic benefit and water use efficiency, respectively.

**Figure 6:**
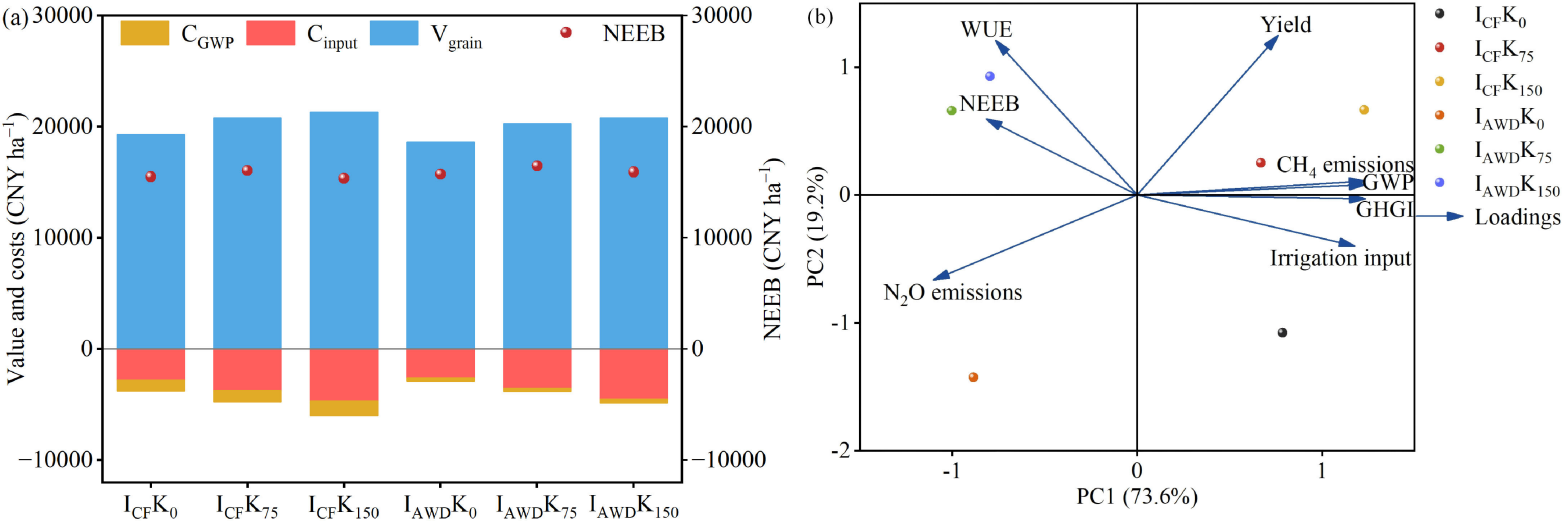
Economic benefit evaluation of each treatment **(A)**. Principal component analysis **(B)**. C_GWP_, C_input_, V_rice_, and NEEB, indicate cost of global warming potential (GWP), cost of input including fertilizer and irrigation, value of rice, and net ecosystem economic benefit, respectively. WUE and GHGI indicate water use efficiency and greenhouse gas intensity, respectively. All traits were mean values of the two years.

Irrigation management and K application alone and combined, did not significantly affect NEEB in either year (**Table 2**). The economic benefit evaluation showed the components of NEEB in each treatment (**Figure 6A**). Although K_150_ had statistically higher value of rice than K_75_, its cost of input especially the fertilizer cost was much higher, leading to a statistically lower NEEB than that of K_75_. I_AWD_ had statistically lower value of rice than I_CF_, however, its cost of GWP, and cost of input especially the irrigation cost were also lower than that of I_CF_, which resulted a statistically higher NEEB than that of I_CF_.

Irrigation management significantly affected irrigation input in two years (**Table 2**). Compared to I_CF_, I_AWD_ decreased irrigation input by 16% and 15% in 2023 and 2024, respectively. Irrigation input was not significantly influenced by K application and its interaction with irrigation management in either year (**Table 2**).

Irrigation management and K application significantly affected WUE (**Table 2**). I_AWD_ elevated WUE by 16% (2023) and 15% (2024), relative to I_CF_, respectively. K_75_ and K_150_ improved WUE by 14%~19% (2023), and 15%~17% (2024), relative to K_0_, respectively. The irrigation management and K application interactions did not significantly influence WUE in either year (**Table 2**).

**Figure 7:**
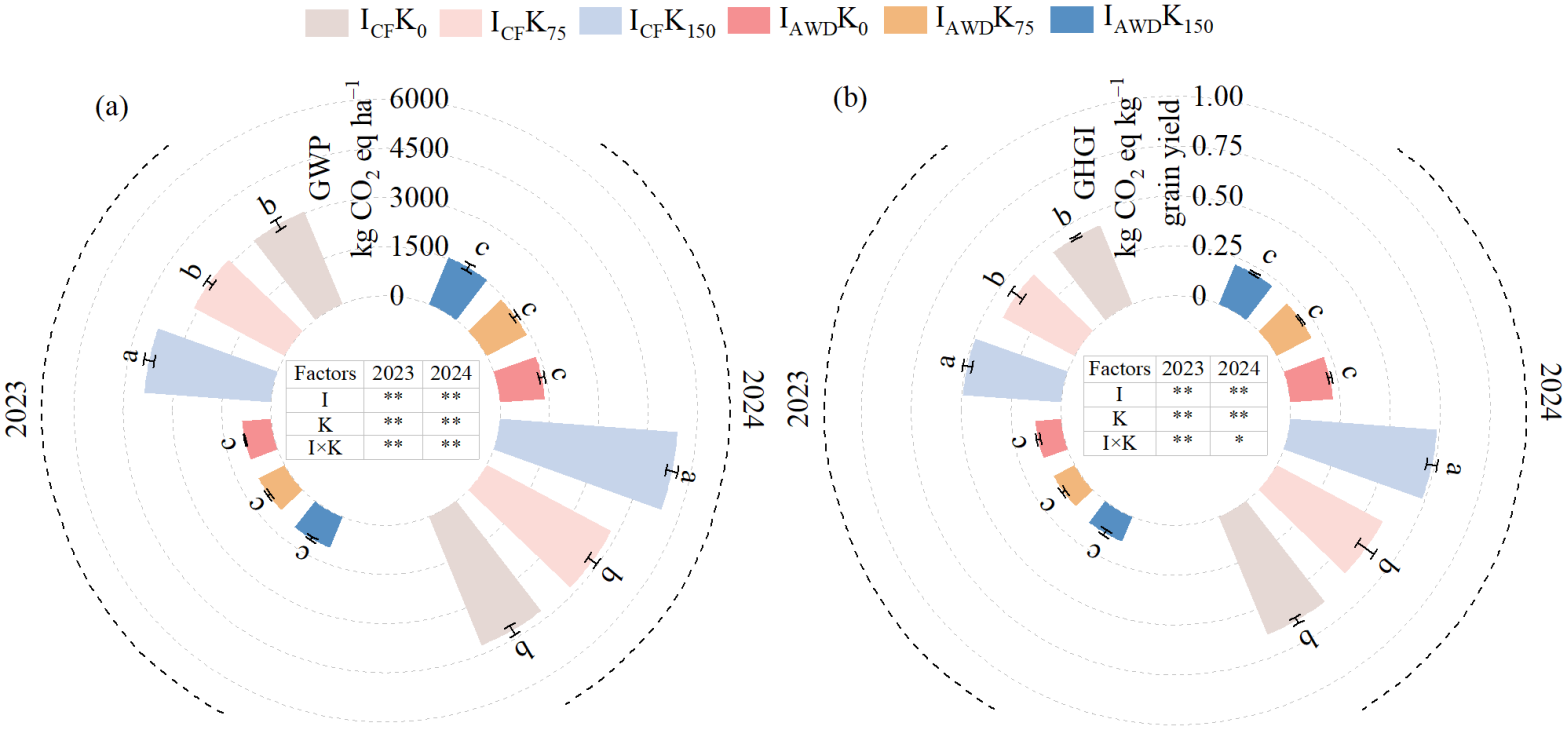
Impacts of irrigation management and K application on global warming potential (GWP) **(A)** and greenhouse gas intensity (GHGI) **(B)** in 2023 and 2024. Different letters above bars indicate significant differences (*p* < 0.05) among treatments. * and ** indicate significant differences at *p* < 0.05 and *p* < 0.01, respectively. Vertical bars indicate mean ± standard deviations (*n* = 3).

Irrigation management, K application, and their interaction significantly affected GWP and GHGI over both years (**Figure 7**). Compared with K_0_, K_150_ elevated GWP by 27% and 28% under I_CF_ in 2023 and 2024, respectively (**Figure 7A**). Under I_CF_, relative to K_0_, K_150_ increased GHGI by 15% in both years (**Figure 7B**). While under I_AWD_, K application did not significantly affect GWP and GHGI in either year (**Figure 7**).

### 3.8 Structural equation modeling and principal component analysis

**Figure 8:**
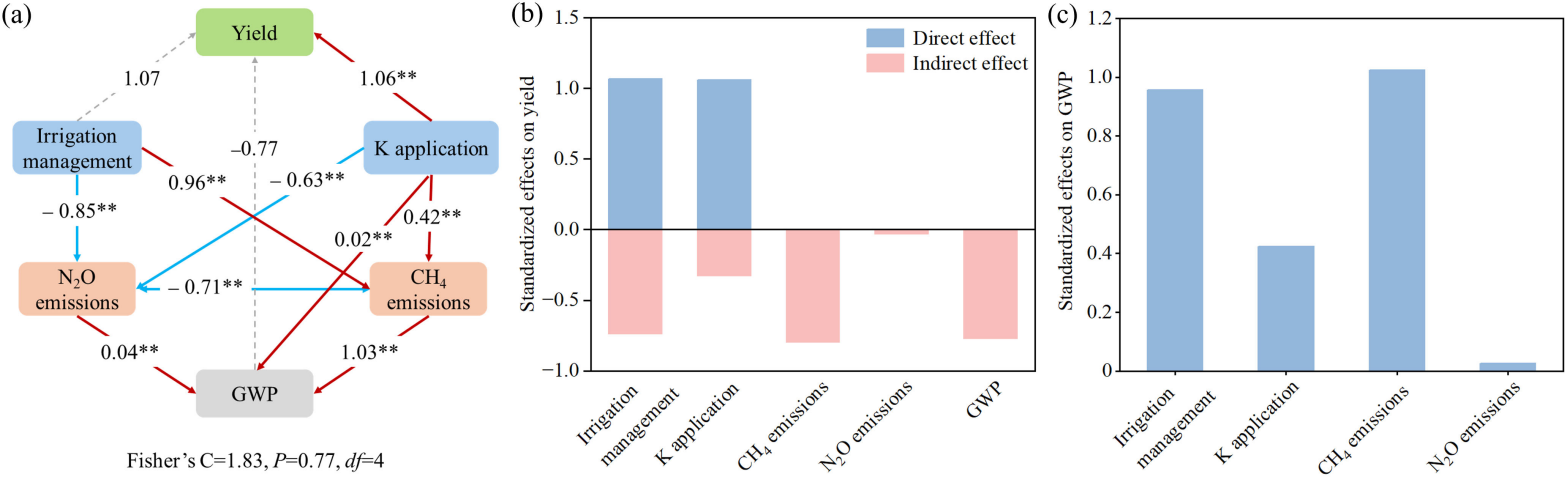
Structural equation modeling showing the impacts of irrigation management, K application, rice yield, cumulative N_2_O and CH_4_ emissions, global warming potential (GWP) **(A)**, the standardized effects on yield of the other indices **(B)**, the standardized effects on GWP of the other indices **(C)**. The model is satisfactorily fitted to the present data, significant paths are illustrated by the gray arrow (no effect), red arrow (positive) and blue arrow (negative) with standardized path coefficients. ** indicates significant at *p* < 0.01.

The SEM showed that rice yield was significantly positively affected by K application (1.06) (**Figure 8**). The cumulative N_2_O emissions were significantly negatively influenced by irrigation management (–0.85) and K application (–0.63). A significantly negative correlation was detected between cumulative CH_4_ and N_2_O emissions (–0.71). Conversely, irrigation management (0.96) and K application (0.42) exerted a significantly positive effect on cumulative CH_4_ emissions. GWP was significantly positively related to both cumulative CH_4_ (0.04) and N_2_O emissions (1.03) (**Figure 8**).

To explore the relationships among the various indicators and treatments, and select the best treatment among all, the PCA was conducted with 8 indicators (cumulative CH_4_ and N_2_O emissions, rice yield, irrigation input, NEEB, WUE, GWP, and GHGI) and 6 treatments (**Figure 6B**). The first two principal components with eigenvalues of 5.89 and 1.54 explain 92.8% of the total variability (**Supplementary Table 5**). PC1 explained 73.6% of the total variance and was associated with cumulative CH_4_ and N_2_O emissions, irrigation input, GWP, and GHGI, and PC1 results indicated that I_AWD_K_0_, I_AWD_K_75_, and I_AWD_K_150_ were regarded as three good treatments considering the 5 indicators. PC2 explained 19.2% of the total variance and included rice yield, WUE and NEEB, and PC2 results indicated that I_AWD_K_75_ and I_AWD_K_150_ were choose as two good treatments. Moreover, I_AWD_K_75_ was closer to NEEB than I_AWD_K_150_, mainly because that K_75_ saved fertilizer cost compared with K_150_ (**Figure 6A**). Thus, I_AWD_ plus 75 kg ha^–1^ K application maintained rice yield while minimizing resources inputs and environmental costs among all the treatments, and was recommended in present study (**Figure 6B**).

## 4 Discussion

### 4.1 Effects of irrigation management and K application on N_2_O emissions

Following each fertilizer application, the elevated inorganic N content supplied an abundant substrate for N_2_O generation (**Table 1**), which might induce a peak in N_2_O flux (**Figures 1A, B**). The lower NH_4_^+^ concentrations under I_AWD_ (by 27% and 20% in TP in 2023 and 2024, respectively, and by 17% and 22% in PP) and the correspondingly higher NO_3_^-^ concentrations under I_AWD_ (by 22% and 25% in TP, and 31% and 22% in PP), reflect a fundamental shift in soil N transformation processes induced by the wetting-drying cycles. The drying phase increases soil aeration, which promotes the transformation of NH_4_^+^ to NO_3_^-^ via nitrification. This interpretation is consistent with findings using ^15^N tracing, where I_AWD_ enhanced N_2_O production primarily through endogenous N mineralized from soil organic matter, with NO_3_^--^derived N_2_O being released rapidly while NH_4_^+^-derived N_2_O exhibited a hysteresis effect due to the time required for NH_4_^+^ conversion to NO_3_^-^ via nitrification (Xu et al., 2020). Furthermore, the accumulation of NO_3_^-^ during the drying phase, combined with the subsequent wetting event that creates transient anoxic microsites, provides ideal conditions for denitrification, during which NO_3_^-^ is reduced to N_2_O when its further reduction to N_2_ is incomplete. In this study I_AWD_ led to a significant cumulative N_2_O emissions increase versus I_CF_ (**Figures 1C, D**). Anaerobic conditions under I_CF_ largely precluded the NO_3_^−^ production from nitrification and promoted denitrification of any NO_3_^−^ to N_2_, together causing lower N_2_O emissions (Verhoeven et al., 2018). The drying periods under I_AWD_ increased nitrification and promoted denitrification, increasing cumulative N_2_O emissions (Devkota et al., 2013). The results were in accordance with Barrat et al. (2022) and Minamikawa. (2025) who emphasized that I_AWD_ caused significant improvements in N_2_O emissions in comparison to I_CF_. Nitrification and denitrification were key microbial processes for N_2_O emissions, and strongly linked to the abundances of *amoA*-AOA, *amoA*-AOB, *nirS*, and *nosZ* genes (Hiis et al., 2024). Consistent with previous studies (Ayiti and Babalola, 2022; Yang et al., 2025), we found that I_AWD_ significantly improved *amoA*-AOA, *amoA*-AOB, *nirK* and *nosZ* gene abundances (**Figures 3A–G**). Regression analysis indicated that these genes abundances were significantly positively related to the N_2_O flux peak, this might explain the significant increase of N_2_O flux peak in I_AWD_ compared to I_CF_. Previous studies had shown that N_2_O flux and cumulative N_2_O emissions were significantly positively related to N-cycling functional genes (Yu et al., 2023; Wu et al., 2025).

The significant increase in soil NH_4_^+^ concentration under K fertilization, particularly in BP where K_75_ and K_150_ increased NH_4_^+^ by 13~24% in 2023 and 12~26% in 2024, also has direct implications for N_2_O emissions. Two potential mechanisms may explain this observation. First, added K^+^ may compete with NH_4_^+^ for soil cation exchange sites, reducing NH_4_^+^ adsorption and thereby increasing NH_4_^+^ availability in the soil solution (Li et al., 2021). Second, K application has been shown to stimulate N_2_O emissions significantly (1.6~10.8 fold) by enhancing the activity of denitrifying microorganisms and acid-resistant nitrifying microorganisms, with denitrification contributing more to the increase (Li et al., 2020). Moreover, the observed promotion of soil NH_4_^+^ concentration by K application provides additional substrate for nitrification, which under alternating oxic-anoxic conditions can fuel both nitrification-derived and coupled nitrification-denitrification derived N_2_O production, as NH_4_^+^ is first oxidized to NO_3_^-^ and then denitrified upon wetting. Recent reviews further indicate that K acts as an essential co-factor for key denitrification enzymes, including nitrate reductase and N_2_O reductase, and modulates the expression of denitrification genes such as *nirS*, *nirK*, and *nosZ*, thereby directly influencing the efficiency of N transformation pathways and the critical N_2_O: N_2_ product ratio (Ayaz et al., 2025). K application hindered the NH_4_^+^ fixation, which further promoted the nitrification process, thereby resulting in substantial inorganic N loss via denitrification-caused N_2_O emissions and NO_3_^-^ leaching (Gao et al., 2022). Nevertheless, K application promoted soil NH_4_^+^ release, and synergistically promoted plant N uptake and N use efficiency, thus decreasing N_2_O emissions (Liu et al., 2024b). Additionally, K application could enhance phloem mediated NO_3_^-^ translocation to roots, and enhance N retention through stimulating NO_3_^−^ immobilization and storage (Xu et al., 2024). K application significantly influenced the N transformation genes abundance (Li et al., 2020). Present study indicated that K application reduced the *amoA*-AOA, *amoA*-AOB, *nirK*, and *nosZ* abundances (**Figure 3**), being consistent with earlier studies (Kielak et al., 2016; Li et al., 2021). Similarly, Ning et al. (2026) suggested that K decreased the genes diversity during nitrification and denitrification processes (e.g. *amoA*/B/C, *nirK*, *nirS*, *nosZ*), suppressed nitrifying and denitrifying bacterial activity, prolonged N retention in the soil, and thereby decreasing gaseous N loss. Our study observed that K application decreased the N_2_O flux peak by reducing the gene abundances of the nitrification and denitrification processes (**Figures 5A–E**). This finding was further supported by that K application significantly negatively correlated with cumulative N_2_O emissions (**Figure 8**).

### 4.2 Effects of irrigation management and K application on CH_4_ emissions

All treatments showed 2~3 peaks, and the maximum CH_4_ flux occurred during the TP in both years (**Figure 2**). In current study, CH_4_ fluxes patterns in I_AWD_ and I_CF_ paddy fields displayed similar characteristics, a finding that aligns with the observations of Islam et al. (2020). Overall, accumulated CH_4_ emissions were greatest in the TP, with lower values in PP. Because of the vigorous growth of rice in the TP, the plant residues and the rice leaf area increased, providing substrates and more channels for CH_4_ emissions (Liao et al., 2023).

Irrigation management emerged as a dominant factor controlling CH_4_ emissions in paddy fields (Kaur et al., 2024). In our research, soil moisture levels in the I_AWD_ treatment remained reduced at the time field water table declined beneath the soil surface, so that I_AWD_ inhibited CH_4_ production and significantly reduced accumulative CH_4_ emissions by 76% averaging in both years, compared to I_CF_ (**Figure 2**), aligning with previous finding (Liu et al., 2024a). Many studies had shown that I_AWD_ increased soil redox potential and reduced soil dissolved organic carbon content (substrate for CH_4_ production), thus reducing CH_4_ emissions (Yao et al., 2014; Ai et al., 2025). CH_4_ production and oxidation were key processes driving soil CH_4_ emission, with their occurrence governed by the activities and abundances of *pmoA* and *mcrA* (Nwokolo and Enebe, 2024). The *mcrA* was typically carried in anaerobic organisms, and I_CF_ created extremely anaerobic conditions in the soil, which was conducive to the methanogens proliferation and activity, thus promoting CH_4_ production (Xu et al., 2023). Because the I_AWD_ frequently created aerobic environments, promoting CH_4_ oxidation by methanotrophs and inhibiting methanogenic CH_4_ production (Yang et al., 2025). Indeed, compared to I_CF_, I_AWD_ significantly decreased *mcrA* and increased *pmoA* gene abundances in our study (**Figure 4**). This corroborates the findings of Yang et al. (2025), who demonstrated that I_AWD_ notably elevated *pmoA* and inhibited *mcrA* gene abundance relative to I_CF_, particularly during soil drying phase.

Present study revealed a significant interaction between irrigation management and K application on cumulative CH_4_ emissions in both years (**Figures 2C, D**). This indicated that the elevated CH_4_ emissions induced by I_CF_ were significantly mitigated by reducing K application from K_150_ to K_75_ (**Figures 2C, D**). Interestingly, I_CF_K_150_ exhibited significantly higher cumulative CH_4_ emission than I_CF_K_0_, while no difference was detected in cumulative CH_4_ emissions between I_AWD_K_150_ and I_AWD_K_0_ (**Figures 2C, D**). On the one hand, K fertilization under I_CF_ promoted aerenchyma tissues development of rice plants (Li et al., 2024b), increased plant residues input (Wang et al., 2024), facilitated organic carbon accumulation (Liang et al., 2025), and improved *mcrA* gene abundance (Jiang et al., 2025). Thus, the adequate supply of water and nutrients significantly increased CH_4_ emissions. On the other hand, aerobic conditions under I_AWD_ reduced *mcrA* and increased *pmoA* gene abundance (Xu et al., 2017). Meanwhile, adequate nutrient supply could alleviate the limitation of water stress on the nutrient absorption of plants (Yeboah et al., 2026). Furthermore, the decreases of CH_4_ emissions caused by I_AWD_ were much larger than that caused by K application. This resulted in similar CH_4_ emissions among the three K applications under I_AWD_.

### 4.3 Effects of irrigation management and K application on yield, NEEB, WUE, GWP, and GHGI

An objective of reducing GHG emissions in paddies was to boost rice yield while achieving a weaker warming effect. I_CF_ generally maintained higher yields than I_AWD_, yet several studies documented insignificant yield variations between I_AWD_ and I_CF_ (Pandey et al., 2014; Bo et al., 2024). when properly managed, I_AWD_ can maintain soil moisture at levels that prevent the yield decline (Chen et al., 2025a). For instance, according to Zhang et al. (2024), mild to moderate I_AWD_ conserved water without compromising rice yield, whereas severe I_AWD_ decrease yield. Here, I_AWD_ reduced the irrigation input without affecting the rice yield, and improved WUE (**Table 2**), aligning with Poddar et al. (2022). Furthermore, studies had also demonstrated that I_AWD_ effectively enhanced rice photosynthetic products accumulation and facilitated carbohydrate translocation to sink organs, leading to stable or increased yield (Cai et al., 2026). Appropriate K application could help alleviate nutrient deficiency induced soil degradation, thereby ensuring the sustainable improvements of rice production (Li et al., 2022a). Compared with K_0_, K fertilization increased rice yield by 10% (Huang et al., 2025). Similarly, K application improved 8%~11% rice yield in our study (**Table 2**). The rice yield and WUE showed no significant increase with rising K application in this study, supporting our previous finding (Li et al., 2022b). K application enhanced WUE by increasing rice grain yield (**Table 2**), which was also demonstrated in the PCA analysis, where K_75_ and K_150_ were closer to rice yield and WUE (**Figure 6B**). .

I_AWD_ increased N_2_O via higher *amoA*-AOA, *amoA*-AOB, *nirK*, and *nosZ* abundances, but reduced CH_4_ via lower *mcrA* abundance and higher *pmoA* abundance (**Figures 3**, **4**); K application decreased N_2_O via lower *amoA*-AOA, *amoA*-AOB, *nirK*, and *nosZ* abundances, but increased CH_4_ via higher *mcrA* abundance. This trade-off ultimately affected the GWP, GHGI and NEEB (**Table 2**; **Figure 7**). Since N_2_O emissions from paddy fields contributed less than 5% to the total GWP, the increase in N_2_O emissions under I_AWD_ was offset by decreased CH_4_ emissions, thereby lowering GWP and GHGI (Chen et al., 2025). Similarly, compared to I_CF_, I_AWD_ reduced accumulative CH_4_ emissions and increased N_2_O emissions, resulting in decreased GWP and GHGI in present study (**Figures 1**, **2**, **7**). In a previous research, Maneepitak et al. (2019) also found that I_AWD_ reduced GWP and GHGI by decreasing CH_4_ emissions and maintaining yield compared to I_CF_. In present study, K application reduced N_2_O emissions and enhanced CH_4_ emissions, and improved GWP (**Figure 7**). Previous study reported that K application increased GWP by improving CH_4_ emissions (Shang et al., 2010). Importantly, the GWP and GHGI of K_150_ were significantly higher than those of K_75_, and K_0_ under I_CF_, but no significant difference in GWP or GHGI was observed among K_150_, K_75_, and K_0_ under I_AWD_. This was due to that I_AWD_ significantly reduced CH_4_ emissions but did not reduce rice yield, thereby reversing the impact of K application on GWP and GHGI under I_AWD_ (**Table 2**). Moreover, the decreased CH_4_ emissions caused by irrigation management far exceeded the increase in CH_4_ emissions resulting from fertilization (Liao et al., 2023). In sum, I_AWD_K_75_ effectively reduced GWP and GHGI while stabilizing rice yield and improving WUE and NEEB (**Figure 6B**).

### 4.4 Limitations of present study

DNA-based qPCR is a well-established, reproducible method for assessing gene abundance, and the results can be directly linked to the potential capacity of microbial communities to perform specific biogeochemical processes. In present study, soil DNA was used to quantify functional gene abundances, which reflects the genetic potential of microbial communities rather than their actual metabolic activity. In contrast, RNA−based analyses (e.g., metatranscriptomics) can capture the expression level of these genes and thus provide a more direct link to instantaneous process rates (Qin et al., 2018; Mise et al., 2025). The discrepancy between gene abundance and expression can be particularly pronounced under fluctuating soil redox conditions during I_AWD_ cycles, where rapid shifts in microbial activity may not be fully reflected in DNA pool sizes (Zhou et al., 2016). Therefore, our results based on DNA−qPCR should be interpreted as an indication of the potential for CH_4_ and N_2_O production and consumption. Future studies are encouraged to combine DNA− and RNA−based approaches to better understand the regulatory mechanisms of GHG under I_AWD_.

qPCR is subject to several inherent limitations: 1) primer bias is a well-recognized issue, different primers may have variable amplification efficiencies across soil microbial DNA templates, resulting in preferential amplification of some sequence variants while underestimating others; 2) even widely used primers, such as the 338F/806R pair employed in this study for 16S rRNA gene amplification, have limited coverage (Morales and Holben, 2009); 3) the use of degenerate primers, although designed to account for sequence variation, can also introduce bias, as sequences with perfect matches to the degenerate sites tend to be preferentially amplified over those with mismatches (Lee et al., 2022). Consequently, the gene copy numbers obtained in this study should be interpreted as relative estimates suitable for comparison among different treatments under identical experimental conditions, rather than as absolute quantitative values. Future studies are encouraged to complement or replace qPCR with PCR−free approaches, to obtain more accurate absolute abundances of functional genes.

While our experiment was conducted in Northeast China, the underlying mechanisms, I_AWD_ shifting soil NH_4_^+^/NO_3_^-^ balance and K application enhancing NH_4_^+^ availability, are universal. Global meta-analyses confirm that I_AWD_ consistently increases N_2_O across diverse climates and soils (Zhao et al., 2024), and K reducing N_2_O have been widely reported in south of China (Li et al., 2020, 2021). Therefore, our mechanistic findings are broadly transferable, though the magnitude of responses may vary with local edaphic and climatic conditions. Multi-region validation is encouraged for future refinement.

The NEEB presented in this study was calculated using fixed market prices for rice and fixed shadow prices for GHG from a specific year. In practice, both commodity prices and carbon prices are subject to considerable interannual and spatial variation. Future studies should incorporate probabilistic economic assessment techniques, such as scenario analysis or Monte Carlo simulation, to better characterize the uncertainty surrounding economic outcomes under I_AWD_ and K fertilization.

In present study the rice root morphological parameters were not measured. However, in rice cultivation, root systems strongly affect N_2_O production and emission. Roots carry nitrification and denitrification microbial communities and provide substrates via exudates. Fan et al. (2026) reported that across five hybrid rice varieties, N_2_O flux was highly significantly positively correlated with root volume and root dry biomass. Root aerenchyma acted as a potential shortcut pathway for N_2_O transport from wetland soils to the atmosphere. Moreover, under I_AWD_, root morphological plasticity can further amplify N_2_O emission. Li et al. (2022c) found that I_AWD_ significantly improved root dry weight, root length, root diameter, and root oxidative activity compared to I_CF_. Therefore, future I_AWD_ and K management studies should routinely quantify root morphological traits to fully capture the rhizosphere component of the N_2_O and CH_4_ emissions.

The long−term sustainability of I_AWD_ combined with K application requires careful consideration of several factors beyond the two−year study period. First, long−term meta-analysis evidence suggests that I_AWD_ had negative effects on soil organic carbon (SOC) over time, with a reported reduction of 5.2% in SOC concentrations and potential depletion of soil organic nitrogen stocks by more than 100 kg N ha^-1^ yr^-1^ (Livsey et al., 2019). These findings indicate that while short−term yield may be sustained, I_AWD_ could gradually reduce soil fertility if practiced continuously without appropriate organic matter amendments. Second, a six−year field study has demonstrated that I_AWD_ significantly exacerbates soil K depletion. Over six years, I_AWD_ decreased soil K balance by 15% compared to I_CF_, suggesting that continuous I_AWD_ without K replenishment could lead to net K mining from soil reserves (Han et al., 2025). These observations highlight a potential tension between short−term agronomic gains from K application and long−term soil K fertility maintenance under I_AWD_.

There are also practical barriers to field-level adoption of I_AWD_. First, the rigid rotational schedules in many irrigation systems leave farmers with little control over the timing of water delivery, further hindering I_AWD_ implementation. Second, most of the fields in such systems are operated by tenants who often lack the authority or risk tolerance to adopt I_AWD_, fearing that any yield reduction, even if unlikely when managed properly. Third, the adoption potential of I_AWD_ varies with seasonal rainfall patterns, farmers are more willing to implement I_AWD_ during the wet season when rainfall provides a safety buffer against drying stress.

## 5 Conclusions

This study examined the impacts of irrigation management and K application on GHG and rice yield. The results revealed that I_AWD_ increased N_2_O emissions due to increasing the abundances of *amoA*-AOA, *amoA*-AOB, *nirK*, and *nosZ* that affecting nitrification and denitrification, and reduced CH_4_ emissions due to decreasing *mcrA* and increasing *pmoA* gene abundances. By contrast, K application significantly lowered N_2_O emissions through reducing *amoA*-AOA, *amoA*-AOB, *nirK*, and *nosZ* genes abundance, and elevated CH_4_ emissions by raising *mcrA* gene abundances. I_AWD_ decreased soil NH_4_^+^ concentration and increased soil NO_3_^-^ concentration, while K application promoted soil NH_4_^+^ concentration without affecting soil NO_3_^-^ concentration. I_AWD_ significantly saved irrigation water without reducing rice yield, and thereby significantly enhancing WUE. K application improved WUE by significantly increasing rice yield. Combined I_AWD_ and K application could significantly decrease GWP and GHGI. SEM analysis indicated that irrigation management and K application had significantly negative effects on N_2_O emissions, and had significantly positive effects on CH_4_ emissions. PCA indicated that I_AWD_K_75_ treatment was closer to WUE and farther from CH_4_ emissions, GWP, GHGI and irrigation input, and thus application of 75 kg K ha^–1^ under I_AWD_ could balance rice productivity and economic benefit as well as environmental issues based on resource-constrained conditions, and was recommended in present study. This study has several limitations. The single-site, two-year design limits broad generalizability, and multi-site validation is needed. DNA-based qPCR reflects genetic potential not actual expression, so that RNA analyses are required for causal inference. NEEB used fixed prices, and sensitivity analysis are needed in future research. These caveats should be considered when applying our findings.

## Data Availability

The raw data supporting the conclusions of this article will be made available by the authors, without undue reservation.

## Supplementary material

Supplementary Figure 1 Daily precipitation and mean temperature at Shenyang, Liaoning, China in 2023 and 2024.

Supplementary Figure 2 Water depth under I_CF_, water depth and soil water potential under I_AWD_ in 2023 and 2024.

Supplementary Table 1 Topsoil (0~20 cm) physicochemical properties before the experiment in 2023.

Supplementary Table 2 Irrigation implementation under I_CF_ and I_AWD_ during the rice growing seasons.

Supplementary Table 3 Primers used in the amplification of genes in present study.

Supplementary Table 4 ANOVAs for genes abundances in 2023 and 2024.

Supplementary Table 5 Variable loading scores for different traits and proportion of variation for each principal component.
