# Supplementary material for: Synergistic effects of AWD irrigation and potassium application on rice yield and paddy field greenhouse gas emissions
Source: Front Plant Sci. 2026 Jun 9;17:1840297. doi: 10.3389/fpls.2026.1840297 (PMC13286799; doi:10.3389/fpls.2026.1840297)
Supplement: Supplementary Figure 1 — Daily precipitation and mean temperature at Shenyang, Liaoning, China in 2023 and 2024. [file SupplementaryFile1.docx]

**Supplementary materials**

**Figures**


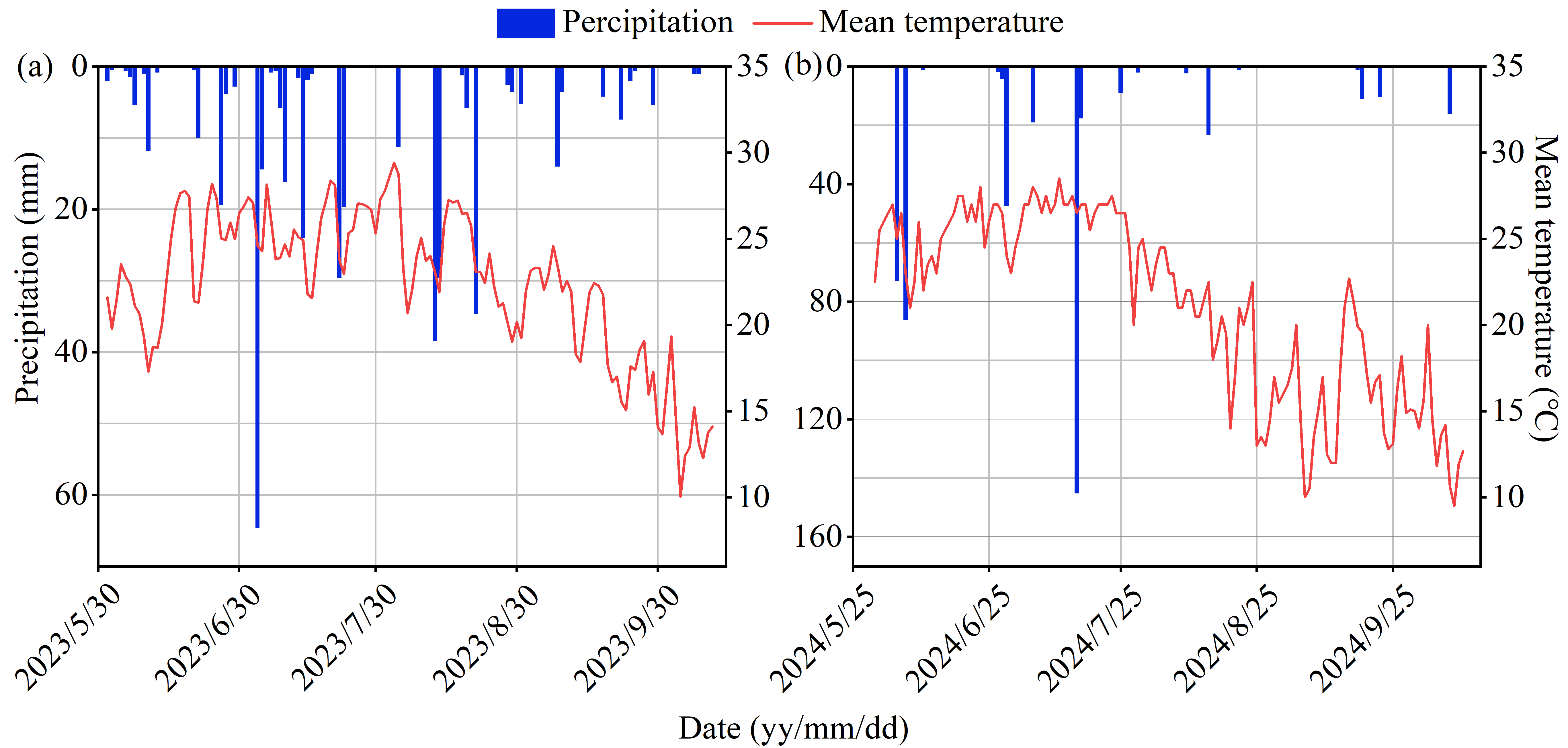


**FIGURE S1**

Daily precipitation and mean temperature during rice growing seasons at Shenyang, Liaoning, China in 2023 and 2024.

**
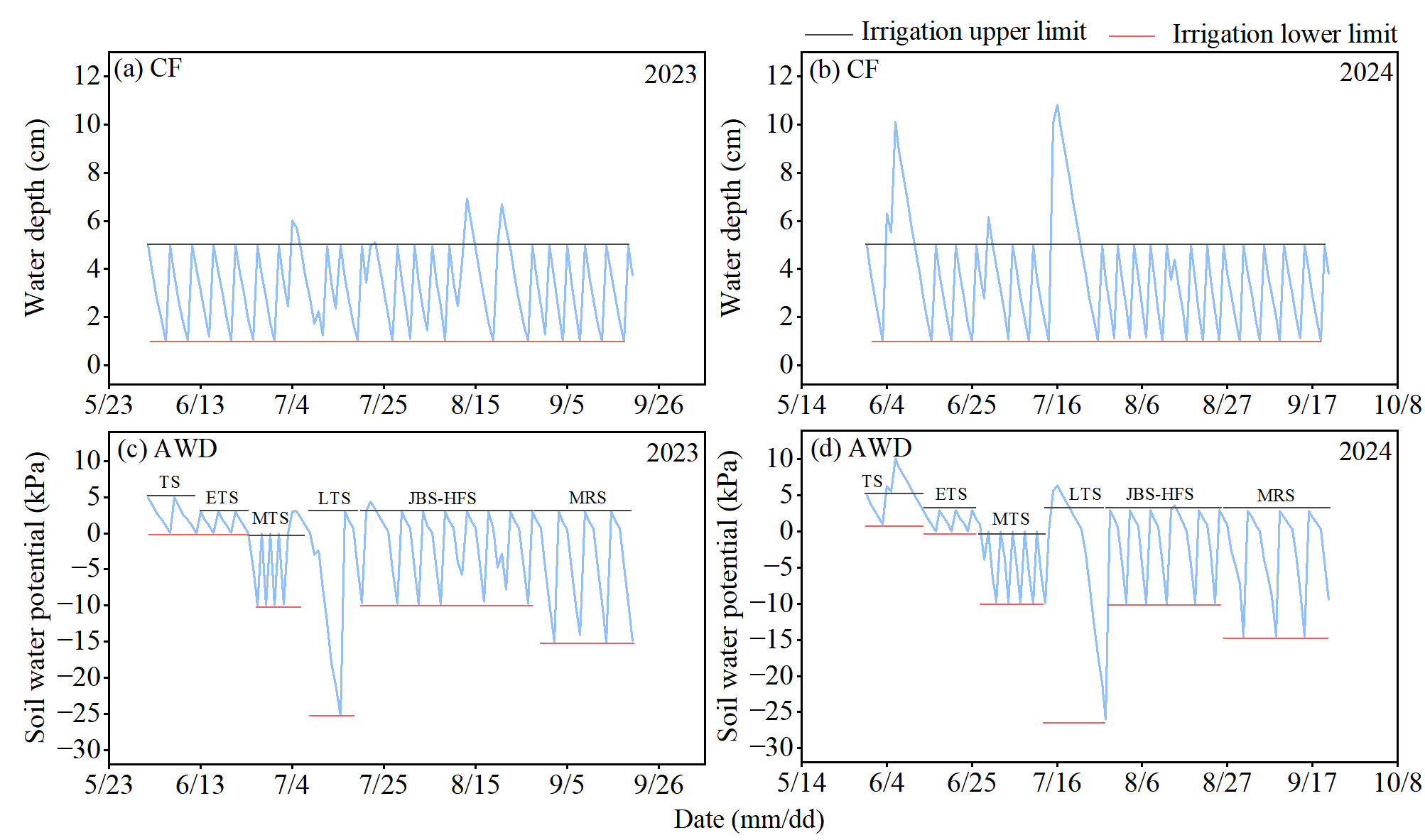
**

**FIGURE S2**

Water depth under I_CF_ during rice growth period in 2023 and 2024. Water depth and soil water potential under I_AWD_ during rice growth period in 2023 and 2024. For I_CF_ (continuous flooding irrigation), the data represent water depth; for I_AWD_ (alternate wetting-drying irrigation), the positive data represent water depth, and negative data represent soil water potential. TS, ETS, MTS, LTS, JBS, HFS, and MRS represent transplanting stage, early tillering stage, middle tillering stage, late tillering stage, jointing-booting stage, heading-flowering stage, milky ripening stage, respectively.

**Tables**

**TABLE S1**

Topsoil (0~20 cm) physicochemical properties before the experiment in 2023

| Topsoil physicochemical properties | |
| --- | --- |
| Sand (%)  Slit (%)  Clay (%)  Bulk density (g cm^−3^) | 24 |
|  | 58 |
|  | 18 |
|  | 1.50 |
| pH | 6.58 |
| Organic mater (g kg^−1^) | 23.2 |
| Total N (g kg^−1^) | 0.91 |
| NH_4_^+^–N (mg kg^−1^) | 10.5 |
| NO_3_^–^–N (mg kg^−1^) | 1.89 |
| Available P (mg kg^−1^) | 24.3 |
| Available K (mg kg^−1^) | 144.6 |

**TABLE S2**

Irrigation implementation of I_CF_ and I_AWD_ during the rice growing seasons.

| Growth stages | I_CF_ |  | I_AWD_ | |
| --- | --- | --- | --- | --- |
|  | Water depth |  | Water depth | Soil water potential thresholds |
| Seedling recovery | 1~ 5 cm |  | 5 cm | 0 kPa |
| Initial tillering stage | 1~ 5 cm |  | 3 cm | 0 kPa |
| Middle tillering stage | 1~ 5 cm |  | 0 cm | –5~ –10 kPa |
| Late tillering stage | 1~ 5 cm |  | 3 cm | –25~ –35 kPa |
| Jointing-booting stage | 1~ 5 cm |  | 3 cm | –5~ –10 kPa |
| Heading-flowering stage | 1~ 5 cm |  | 3 cm | –5~ –10 kPa |
| Milky ripening stage | 1~ 5 cm |  | 3 cm | –10~ –20 kPa |
| Yellow ripening stage | Natural drying | | | |

I_CF_ and I_AWD_ represent continuous flooding irrigation and alternate wetting-drying irrigation, respectively.

**TABLE S3**

Primers used in the amplification of genes in present study.

| Gene | Primer | Primer sequence (5' to 3') | Reference |
| --- | --- | --- | --- |
| *amoA-*AOA | Arch-amoA26F | GACTACATMTTCTAYACWGAYTGGGC | Park et al. 2008 |
|  | Arch-amoA417R | GGKGTCATRTATGGWGGYAAYGTTGG |  |
| *amoA-*AOB | amoA1F | GGGGTTTCTACTGGTGGT | Rotthauwe et al. 1997 |
|  | amoA2R | CCCCTCKGSAAAGCCTTCTTC |  |
| *nirK* | nirKF1aCuF | ATCATGGTSCTGCCGCG | Throbäck et al. 2004 |
|  | nirKR3aCuR | GCCTCGATCAGRTTGTGGTT |  |
| *nirS* | cd3aF | GTSAACGTSAAGGARACSGG | Throbäck et al. 2004 |
|  | R3cdR | GASTTCGGRTGSGTCTTGA |  |
| *nosZ* | nosZ-1126F | GGGCTBGGGCCRTTGCA | Chen et al. 2012 |
|  | nosZ-1381R | GAAGCGRTCCTTSGARAACTTG |  |
| *mcrA* | mcrA-F | GGTGGTGTMGGATTCACACARTAYGCWACAGC | Barbier et al. 2012 |
|  | mcrA-R | TTCATTGCRTAGTTWGGRTAGTT |  |
| *pmoA* | A189F | GGNGACTGGGACTTCTGG | Costello and Lidstronm 1999 |
|  | mb661R | CCGGMGCAACGTCYTTACC |  |

**TABLE S4**

ANOVAs for genes abundances in 2023 and 2024.

| Indicators | 2023 | | |  | 2024 | | |
| --- | --- | --- | --- | --- | --- | --- | --- |
|  | I | K | I×K |  | I | K | I×K |
| *amoA*-AOA | * | * | ns |  | * | * | ns |
| *amoA*-AOB | * | * | ns |  | * | ** | ns |
| *nirK* | * | * | ns |  | ** | ** | ns |
| *nirS* | ns | ns | ns |  | ns | ns | ns |
| *nosZ* | * | * | ns |  | * | * | ns |
| *mcrA* | ** | * | ns |  | * | * | ns |
| *pmoA* | ** | ns | ns |  | * | ns | ns |

I represents irrigation management, and K represents potassium application. * and ** indicate signiﬁcance level at *p* ≤ 0.05 and *p* ≤ 0.01, respectively. ns indicates non-signiﬁcant.

**TABLE S5**

Variable loading scores for different traits and proportion of variation for each principal component

| Traits | PC1 | PC2 |
| --- | --- | --- |
| Cumulative N_2_O emissions | **-0.37** | -0.33 |
| Cumulative CH_4_ emissions | **0.41** | 0.05 |
| Irrigation input | **0.39** | -0.20 |
| Rice yield | 0.25 | **0.62** |
| Water use efficiency (WUE) | -0.26 | **0.60** |
| Global warming potential (GWP) | **0.41** | 0.04 |
| Greenhouse gas intensity (GHGI) | **0.41** | -0.02 |
| Net ecosystem economic benefit (NEEB) | -0.27 | **0.30** |
| Eigenvalues | 5.89 | 1.54 |
| Variability (%) | 73.6 | 19.2 |
| Cumulative variability (%) | 73.6 | 92.8 |

The bold text indicates the principal component.

**References**

Barbier, B.A., Dziduch, I., Liebner, S., 2012. Methane-cycling communities in a permafrost-affected soil on Herschel Island, Western Canadian Arctic: active layer profiling of mcrA and pmoA genes. FEMS Microbiol. Ecol. 82 (2), 287–302

Chen, Z., Liu, J., Wu, M., Xie, X., Wu, J., Wei, W., 2012. Differentiated response of denitrifying communities to fertilization regime in paddy soil. Microb Ecol. 63: 446–459. https://doi.org/10.1007/s00248-011-9909-5

Costello, A.M., Lidstrom M.E., 1999. Molecular characterization of functional and phylogenetic genes from natural populations of methanotrophs in lake sediments. Appl. Environ. Microbiol. 65 (11), 5066-5074

Park, S.J., Park, B.J., Rhee, S.K., 2008. Comparative analysis of archaeal 16s rRNA and amoA genes to estimate the abundance and diversity of ammonia-oxidizing archaea in marine sediments. Extremophiles. 12, 605–615. https://doi.org/10.1007/s00792-008- 0165-7

Rotthauwe, J.H., Witzel K.P., Liesack, W., 1997. The ammonia monooxygenase structural gene amoA as a functional marker: molecular fine-scale analysis of natural ammonia-oxidizing populations. Appl. Environ. Microbiol. 63 (12), 4704–4712. https://doi.org/10.1128/aem.63.12.4704-4712.1997

Throbäck, I.N., Enwall, K., Jarvis, Å., Hallin, S. 2004. Reassessing PCR primers targeting nirS, nirK and nosZ genes for community surveys of denitrifying bacteria with DGGE. FEMS Microbiol. Ecol. 49 (03), 401-417. https://doi.org/10.1016/j.femsec.2004.04.011
